# [^11^C]PBR28 MR–PET imaging reveals lower regional brain expression of translocator protein (TSPO) in young adult males with autism spectrum disorder

**DOI:** 10.1038/s41380-020-0682-z

**Published:** 2020-02-19

**Authors:** N. R. Zürcher, M. L. Loggia, J. E. Mullett, C. Tseng, A. Bhanot, L. Richey, B. G. Hightower, C. Wu, A. J. Parmar, R. I. Butterfield, J. M. Dubois, D. B. Chonde, D. Izquierdo-Garcia, H. Y. Wey, C. Catana, N. Hadjikhani, C. J. McDougle, J. M. Hooker

**Affiliations:** 1 Department of Radiology, Athinoula A. Martinos Center for Biomedical Imaging, Massachusetts General Hospital, Charlestown, MA USA; 2 Harvard Medical School, Boston, MA USA; 3 Lurie Center for Autism, Massachusetts General Hospital, Lexington, MA USA; 4 Gillberg Neuropsychiatry Center, University of Gothenburg, Sahlgrenska Academy, Gothenburg, Sweden

**Keywords:** Molecular biology, Neuroscience, Autism spectrum disorders

## Abstract

Mechanisms of neuroimmune and mitochondrial dysfunction have been repeatedly implicated in autism spectrum disorder (ASD). To examine these mechanisms in ASD individuals, we measured the in vivo expression of the 18 kDa translocator protein (TSPO), an activated glial marker expressed on mitochondrial membranes. Participants underwent scanning on a simultaneous magnetic resonance–positron emission tomography (MR–PET) scanner with the second-generation TSPO radiotracer [^11^C]PBR28. By comparing TSPO in 15 young adult males with ASD with 18 age- and sex-matched controls, we showed that individuals with ASD exhibited lower regional TSPO expression in several brain regions, including the bilateral insular cortex, bilateral precuneus/posterior cingulate cortex, and bilateral temporal, angular, and supramarginal gyri, which have previously been implicated in autism in functional MR imaging studies. No brain region exhibited higher regional TSPO expression in the ASD group compared with the control group. A subset of participants underwent a second MR–PET scan after a median interscan interval of 3.6 months, and we determined that TSPO expression over this period of time was stable and replicable. Furthermore, voxelwise analysis confirmed lower regional TSPO expression in ASD at this later time point. Lower TSPO expression in ASD could reflect abnormalities in neuroimmune processes or mitochondrial dysfunction.

## Introduction

Autism spectrum disorder (ASD) is behaviorally defined by the presence of deficits in social communication, and the presence of repetitive behaviors and restricted interests [1]. ASD, which affects 1 in 59 children [2], is characterized by phenotypic and genetic heterogeneity, and likely has several etiologies. Among candidate mechanisms for the underlying neuropathology are neuroimmune mechanisms as well as mitochondrial dysfunction, which have been repeatedly associated with ASD [3–5].

Neuroimmune mechanisms are strongly implicated in the etiology of at least one type of ASD. This stems from transcriptomic analyses that implicate genes involved in innate immunity, and microglial and astrocytic markers in ASD [5, 6]. In addition, epidemiological work has repeatedly associated maternal immune response to infection with an increased risk for ASD in offspring [7–9], with recent work showing that hospitalization due to maternal infection during pregnancy increases the risk for autism in the offspring by almost twofold [10]. Furthermore, elevated maternal cytokines, chemokines, and other inflammatory markers, such as elevated maternal C-reactive protein (a marker of systemic inflammation), have been associated with autism in the offspring [11, 12]. Individuals with ASD also have a greater number of family members with autoimmune disorder than controls [13, 14], and the presence of certain autoimmune disorders in the mother has been linked with a significantly higher risk of having a child with autism [15]. In individuals with ASD, abnormal levels of cytokines, particularly increases in interleukin-1beta (IL-1β), IL-6, IL-8, interferon-gamma (IFN-γ), monocyte chemotactic protein-1 (MCP-1), and eotaxin, as well as a decrease in transforming growth factor-β1, have been reported and may be associated with ASD symptom severity (see meta-analysis and review by Masi et al. [16]).

The neuroimmune mechanisms have been supported through studies in children with autism. For example, proinflammatory response/T-cell activation upon mononuclear cell stimulation is associated with a more impaired behavioral phenotype [17]. In addition, a small clinical trial reported symptom improvement in children with autism using celecoxib, a cyclooxygenase-2 (COX-2)-selective nonsteroidal anti-inflammatory drug, which was administered as adjunctive treatment to risperidone [18].

Mitochondrial dysfunction has been repeatedly associated with ASD (reviewed in refs. [19, 20]). Abnormal biomarkers of mitochondrial dysfunction are not only observed in individuals with mitochondrial disease and ASD, but also in the general population of ASD where mitochondrial dysfunction is estimated to be about 500× more prevalent than in the general population (5.0% vs. 0.01%) [19]. More than a dozen studies have reported mitochondrial dysfunction in ASD (reviewed in ref. [20]), including higher levels of oxidative damage to mitochondrial proteins/abnormal mitochondrial protein levels in the frontal lobe, temporal lobe, and cerebellum in ASD [4, 21, 22].

Based on the collective evidence, the investigation of neuroimmune and mitochondrial dysfunction-related mechanisms in vivo in adults with ASD is warranted. Translocator protein (18 kDa) (TSPO), a mitochondrial protein expressed on microglia and astrocytes, has been implicated in several physiological processes, including immune modulation and mitochondrial homeostasis. TSPO has been suggested as potential endophenotype and therapeutic target for psychiatric disorders [23], and can be imaged in vivo using positron emission tomography imaging (PET). So far, TSPO in individuals with ASD has only been investigated in one PET study with [^11^C]PK11195 [24], and one very small post mortem study [25], which come inherent with the limitation of older-generation PET radiotracers (including high nonspecific binding and poor signal to noise) and post mortem work (including small sample size, despite pooling across sex and age groups, due to limited availability of brain tissue). In vivo PET imaging of TSPO with a second-generation radiotracer such as [^11^C]PBR28 is therefore a promising tool to investigate TSPO in ASD. TSPO imaging is multifaceted as there are several mechanisms, including neuroimmune and mitochondrial function-related processes that can alter TSPO expression in a disease-specific context, with little being known in the context of ASD.

Furthermore, in order to address the question of variability in TSPO expression across the autism spectrum and potential association with symptom severity, this work includes individuals with varying levels of functioning and disease severity. To date, low-functioning adults with ASD have been underrepresented in PET neuroimaging studies [26], and recent calls to action have been made in order for autism research to fully represent autism across the disease spectrum [27, 28]. Compared with controls, individuals with ASD exhibit upregulation in microglial and astrocytic genes [5], for which higher levels of expression have been reported in typically developing males compared with females [29]. This is particularly interesting considering the higher prevalence of ASD in males, and suggests that neuroimmune mechanisms should be investigated separately in males and females with ASD. Given that ASD is a chronic and relatively stable condition in adulthood, we also aimed to determine in a longitudinal design whether TSPO expression is stable across a period of several months in individuals with ASD. A lack of stable TSPO expression, despite stable symptoms of ASD, would suggest that TSPO varies independently of the symptoms of ASD.

The study aims were to assess whether young adult males with autistic disorder exhibit abnormal in vivo brain expression of TSPO using [^11^C]PBR28 PET imaging, and to determine whether TSPO expression is stable over a period of several months in both males with ASD and typically developing peers.

## Materials and methods

The study was conducted at Massachusetts General Hospital. The protocol was approved by the Institutional Review Board of Partners Healthcare and the Radioactive Drug Research Committee. Participants or their surrogate, when appropriate, provided written informed consent. In situations where surrogate consent was obtained, individuals with ASD also provided assent.

### Study participants

Fifteen male participants with ASD (24.1 years ± 5.5 (mean ± SD)) and 18 typically developing age-matched male control participants (CON) (25.5 years ± 5.8) were included in the data analysis. In order to conduct the work in a homogeneous group, and given the reported sex differences in glial biology and autism, only males were included in this study. All participants were recruited at the Lurie Center for Autism and underwent simultaneous magnetic resonance (MR)–PET scans at the Athinoula A. Martinos Center for Biomedical Imaging. To meet inclusion criteria, participants had to be males between 18 and 40 years of age, present a mixed or high binding affinity for [^11^C]PBR28 (based on the Ala147Thr TSPO polymorphism, see below), not be taking any anti-inflammatory drugs, immune-modulating drugs, or benzodiazepines, be nonsmokers, and have an intelligence quotient (IQ) at or above the range of moderate intellectual disability (>35) for individuals with ASD and normal IQ, i.e., an IQ ≥ 85 for CON in order to exclude controls with other neurodevelopmental disorders. All participants were also required to not be taking any illicit drugs (verified by a urine drug test at the day of enrollment and day of scan) and have no MR–PET safety contraindications. Individuals with ASD with epilepsy were excluded if they had a seizure or change in seizure medication in the last 6 months, as epilepsy is associated with an increase in [^11^C]PBR28 uptake [30]. Fourteen participants with ASD had no epilepsy, and one participant had occasional fever-associated petit mal seizures in early childhood. Data were analyzed with and without this individual, and the main findings remained unchanged. One additional individual with ASD was enrolled and scanned, but not included in data analysis, because he did not complete the 30-min PET scan. Eight individuals with ASD and ten CON underwent a second [^11^C]PBR28 MR–PET scan in order to assess the stability of TSPO expression over several months.

#### Genotyping for TSPO polymorphism and controlling for PBR28 binding affinity differences

As is the case for all second-generation TSPO radioligands, a single-nucleotide polymorphism on exon 4 of the TSPO gene, rs6971, which results in an alanine-to-threonine substitution, confers differential binding affinity to [^11^C]PBR28. Thus, participants were genotyped for the Ala174Thr TSPO polymorphism, and only individuals with C/C genotype (i.e., Ala/Ala, high-affinity binders (HAB)) and C/T genotype (i.e., Ala/Thr, mixed-affinity binders (MAB)) were included. At screening, three controls and three individuals with ASD were found to have the T/T genotype, which confers low binding affinity and negligible PET signal [31], and were therefore not scanned. In order to account for individual differences in global signal, after ensuring that there were no differences in whole-brain mean between ASD and CON groups, standardized uptake values (SUV) were normalized to whole-brain mean at the subject level, as done previously for [^11^C]PBR28 [32–40]. Furthermore, TSPO genotype was added as a regressor of noninterest in voxelwise analyses.

#### Autism diagnosis and clinical characterization

Individuals diagnosed with autistic disorder based on the DSM-IV-TR [41] by a board-certified psychiatrist with experience in the diagnosis of ASD were included. Autism diagnosis was corroborated by the Autism Diagnostic Interview-Revised (ADI-R) [42, 43] and the Autism Diagnostic Observation Schedule-2 (ADOS-2), module 4 [44] (except for one participant who was assessed with the ADOS, module 4) [45]. All participants also met criteria for ASD based on the DSM-5 [1]. IQ was determined with the abbreviated IQ score on the Stanford Binet Intelligence Scales, Fifth Edition [46].

### PET radiotracer synthesis

[^11^C]PBR28 was synthesized by the Martinos Center Radiotracer Production Lab for immediate use in MR–PET imaging. In short, the desmethyl precursor (1 mg in 100 µl of *N,N-*dimethylformamide) is loaded into a 5-ml stainless-steel loop for reaction with ^11^CH_3_I (8 ccm N_2_ as a carrier) using the Wilson captive solvent method. After a 3-min in-loop reaction time, [^11^C]PBR28 is purified by reversed-phase chromatography (Gemini-NX C18 semipreparative column using an isocratic elution with MeCN:aqueous ammonium formate, 70:30) and reformulated by solid-phase extraction in 10% ethanol/saline and then aseptically filtered. The final product is assayed in a series of quality control experiments prior to release for human imaging.

### MR–PET data acquisition

In order to help reduce anxiety and minimize motion, all participants underwent a training protocol that included viewing videos demonstrating in-house procedures associated with a simultaneous MR–PET scan, and undergoing a training scan in the real scanner. For a description of the training protocol, including videos, see ref. [47]. Participants were scanned using a hybrid MR–PET scanner, the Siemens BrainPET, which is based on a head-only PET camera that fits into the bore of a 3 Tesla TIM Trio MR scanner [48]. *MR data acquisition:* an eight-channel MR head receive coil was used, and a high-resolution anatomical scan, a multi-echo magnetization-prepared rapid acquisition gradient echo with prospective motion correction (using EPI-based volumetric navigators, vNavs), with TR = 2530 ms, TE[1–4] = 1.66, 3.53, 5.4, and 7.27 ms, FOV = 280 mm, flip angle = 7°, and voxel size = 1 mm isotropic was acquired [49]. *PET data acquisition and image reconstruction:* [^11^C]PBR28 was administered to participants outside of the scanner as a slow bolus injection by a licensed nuclear medicine technologist through an intravenous catheter in the antecubital vein of the arm. Mean injected dose and mass did not differ across ASD and CON groups at either time point, or within groups across the two time points (all *p* > 0.05). Participants underwent ~1 h of simultaneous MR–PET imaging. Attenuation correction was conducted using a validated MR-based methodology with statistical parametric mapping-based, pseudo-computed tomography methodology [50, 51]. PET data were reconstructed using the three-dimensional ordinary Poisson-ordered subset expectation maximization algorithm from prompt and random coincidences, normalization, attenuation, and scatter coincidence sinograms using 32 subsets and 1 iteration. The reconstructed volume consisted of 153 slices with 256 × 256 pixels and 1.25 × 1.25 × 1.25 mm^3^.

### PET data analysis

To minimize the impact of subject motion, the emission data collected from 60 to 90 min post radioligand injection were divided into six 5-min frames. The corresponding SUV images were separately generated, co-registered (using the MCFLIRT tool [52] available in FSL version 5.0.7, https://fsl.fmrib.ox.ac.uk/fsl), and averaged to obtain a mean SUV image for each participant (SUV_60–90_). The SUV_60–90_ was then registered to the structural scan using FreeSurfer’s (https://surfer.nmr.mgh.harvard.edu) spmregister, and to Montreal Neurological Institute (MNI) space by applying a transformation matrix to skull-stripped SUV images, which was calculated from the FSL FLIRT and FNIRT registration of the structural T1 image to MNI standard space. The brain mask used for skull stripping was based on a combination of all anatomical regions obtained from FreeSurfer’s automated parcellation and segmentation, based on the Desikan/Killiany atlas using FreeSurfer version 6.0. For each individual, skull-stripped images were normalized by the whole-brain mean not including the ventricles (SUVR_60–90_), and spatially smoothed with a Gaussian kernel of 8 mm full width at half maximum.

### Between-group difference in [^11^C]PBR28 SUVR

Voxelwise comparisons were conducted between ASD and CON groups at both time points. *Controlling for atrophy****:*** partial volume correction (PVC) was conducted in order to account for partial volume effects using region-based PVC with the geometric transfer matrix method available in the PETSurfer PVC pipeline from FreeSurfer. To ensure that potential differences in volumes of brain areas were not driving group differences in [^11^C]PBR28 uptake, the volume of the regions with a difference in voxelwise [^11^C]PBR28 binding across groups was assessed in subject space and normalized by total intracranial volume using FreeSurfer. ASD subgroup analyses were conducted to compare individuals with high-functioning autism (HFA) with IQ ≥ 85 vs. CON and low-functioning autism (LFA) with IQ < 85 vs. CON, as well as HFA vs. LFA.

### Long-term stability of [^11^C]PBR28 SUVR

For the eight ASD and ten CON who underwent a second [^11^C]PBR28 MR–PET scan, SUVR_60–90_ was assessed for 14 regions of interest (ROIs) and percent signal change, and Pearson’s *r* was calculated between SUVR_60–90_ at those two time points. Furthermore, to assess potential bias, Bland–Altman plots were created and the coefficient of repeatability (CR) was calculated as 1.96 × SD of the bias [53]. The ROIs included composite ROIs for the frontal, parietal, temporal, and occipital lobes, as well as ROIs for the insula, cingulate, caudate, putamen, pallidum, thalamus, hippocampus, amygdala, cerebellum, and white matter with bilateral ROIs merged together, as done previously [38]. Thirteen ROIs were defined according to the Automated Anatomical Labeling human brain atlas distributed with PMOD version 5.4 (PMOD Technologies LLC, Zurich, Switzerland), and the white matter ROI was based on the tissue maps in PMOD, thresholded to 98% probability.

### Statistics

Voxelwise analyses using a general linear model were performed in FSL using randomise for nonparametric statistics, with threshold-free cluster enhancement and 10,000 permutations, with familywise error rate (FWE) to correct for multiple comparisons (*p*_FWE_ < 0.05) [54]. For subgroup analyses of LFA vs. HFA and the subgroup analysis of ASD vs. CON patients who underwent a second scan, parametric *t* tests were conducted using FSL FEAT with mixed effects and ordinary least squares, and a statistical threshold of *Z* > 2.3, *p*_cluster_ < 0.05 [55]. For all voxelwise analyses, TSPO genotype and age were entered as nuisance regressors. Bland–Altman plots were created in R using the BlandAltmanLeh package. All other statistical tests, including two-way analysis of variance (ANOVA), Pearson’s *r*, Mann–Whitney *U* tests for continuous variables, and chi-square test for categorical variables, were conducted in GraphPad Prism version 7.0 (GraphPad Software, La Jolla, CA).

## Results

### Participant characteristics

ASD and CON groups did not differ significantly in age, sex, TSPO genotype distribution, or radiochemical measures. As expected based on our study design, the ASD group had lower IQ than the CON group (*p* < 0.0001). Furthermore, participants with ASD had slightly higher body mass index (BMI) than CON (*p* = 0.03). See Table 1 for participant demographic information, cognitive and neuropsychiatric characteristics, prescribed medication, and radiochemical measures.

**Table 1:** Characteristics for males with autism spectrum disorder (ASD) and male controls (CON).

	ASD	CON	ASD	CON	*p* value
	Scan 1	Scan 1	Scan 2	Scan 2	ASD vs. CON scan 1
	*N* = 15	*N* = 18	*N* = 8	*N* = 10	
Demographics					
Sex	100% male	100% male	100% male	100% male	
Age	24.1 ± 5.5	25.5 ± 5.8	23.4 ± 4.6	28.4 ± 6.2	*U* = 113*, p* = 0.44, ns
TSPO genotype C/C vs. C/T (i.e., number of HAB/MAB)	8/7	13/5	4/4	5/5	*X*^2^ = 1.26, *p* = 0.26, ns
BMI	28.9 ± 5.9	24.7 ± 3.6	29.3 ± 6.8	25.7 ± 3.8	*U* = 74*, p* = 0.03
Neuropsychiatric scores					
IQ	86.1 ± 19.2	110.3 ± 11.4	95.9 ± 11.1	110.2 ± 12.2	*U* = 28*, p* < 0.0001
ADOS-2_Social affect_	12.6 ± 3.5	–	11.4 ± 2.7	–	
ADOS-2_Restricted repetitive behaviors_	4.2 ± 1.3	–	4.4 ± 1.5	–	
ADOS-2_Total (Social affect + Restricted repetitive behaviors)_	16.8 ± 4.1	–	15.8 ± 3.2	–	
ADI-R_Social interaction_	22.7 ± 4.1	–	22.0 ± 4.9	–	
ADI-R_Communication_	18.1 ± 3.0	–	16.9 ± 3.3	–	
ADI-R_Restricted, repetitive, and stereotyped patterns of behavior_	6.7 ± 1.8	–	6.4 ± 2.1	–	
ADI-R_Abnormality of development_	4.1 ± 1.3	–	4.0 ± 1.6	–	
CGI	4.6 ± 0.7	1 ± 0	4.4 ± 0.7	1 ± 0	*U* = 0*, p* < 0.0001
PET measures					
Injected radioactivity (mCi)	14.1 ± 0.5	13.8 ± 1.2	13.7 ± 1.3	13.9 ± 1.2	*U* = 130*, p* = 0.87, ns
Specific activity (mCi/nmol)	1.9 ± 0.8	1.9 ± 0.7	1.4 ± 0.8	2.0 ± 0.5	*U* = 124*, p* = 0.71, ns
Mass injected (μg)	2.6 ± 0.2	2.5 ± 0.6	3.3 ± 0.5	2.4 ± 0.8	*U* = 126.5, *p* = 0.77, ns
Pharmacological drugs					
Antidepressants	6	0	2	0	
Anxiolytics	5	0	3	0	
Nonstimulant cognitive enhancers	3	0	1	0	
Atypical antipsychotics	3	0	1	0	
Anticonvulsants	1	0	1	0	
Stimulants	1	0	1	0	

Data are presented as mean ± SD. For pharmacological drugs, the *N* number indicates the number of participants taking a drug of a given class. ADOS-2 total was calculated as the sum of the two ADOS-2 domains: ADOS-2 social affect and ADOS-2 restricted repetitive behaviors.

*HAB* high-affinity binders, *MAB* mixed-affinity binders, *BMI* body mass index, *IQ* intelligence quotient, *ADOS-2* autism diagnostic observation schedule-2, *ADI-R* autism diagnostic interview revised, *CGI* clinical global impression, *χ*^2^ Chi-square, *U* Mann–Whitney U, *ns* not significant.

### Between-group difference in [^11^C]PBR28 SUVR

A two-way ANOVA with group (ASD vs. CON) and TSPO genotype (CC vs. CT, i.e., HAB vs. MAB) on whole-brain [^11^C]PBR28 SUV calculated in subject space showed the predicted main effect of genotype (*F*(1,29)=8.63, *p* < 0.05) with higher SUV in HAB than MAB, but no main effect of group and no interaction between group and TSPO genotype. Therefore, whole-brain normalization was performed, as previously done for [^11^C]PBR28 [32–40].

**Fig. 1:**
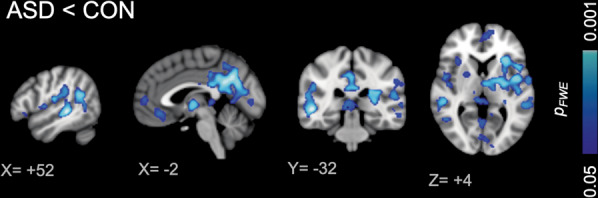
Statistical difference in [^11^C]PBR28 SUVR_60–90_ between ASD and CON groups. Bilateral insular cortex, putamen, precuneus/posterior cingulate cortex, orbitofrontal cortex, lateral occipital cortex, superior temporal gyrus, angular gyrus, supramarginal gyrus, and the left postcentral gyrus showed lower relative TSPO expression in ASD compared with CON. There was no brain region, which showed higher relative TSPO expression in ASD compared with CON.

Between-group voxelwise analysis showed that [^11^C]PBR28 binding was significantly lower in the bilateral insular cortex, putamen, precuneus/posterior cingulate cortex, orbitofrontal cortex, lateral occipital cortex, superior temporal gyrus, angular gyrus, supramarginal gyrus, and the left postcentral gyrus in ASD compared with CON (*p*_FWE_ < 0.05, Fig. 1). No area showed a higher signal in individuals with ASD compared with CON. Furthermore, several months later, the participants with ASD, who underwent a second scan, showed lower TSPO expression compared with CON in a subset of the regions, which showed decrease in the larger group, confirming the stability of lower TSPO expression in ASD (*Z* > 2.3, *p*_cluster_ < 0.05, Fig. S1).

#### Controlling for atrophy

PVC SUVR_60–90_ confirmed a significant decrease in TSPO signal in subject space (Fig. S2). ASD and CON groups did not differ in terms of the volume of the post hoc mask in subject space normalized by ICV, suggesting that the decrease was not an artifact due to atrophy of those structures in ASD (Fig. S3).

#### Low-functioning vs. high-functioning ASD subgroups

Analyses of LFA vs. CON and HFA vs. CON showed that both the LFA subgroup (*N* = 6, mean IQ ± SD: 61.8 ± 12.5) and the HFA subgroup (*N* = 9, 98 ± 9.0) showed decreased regional TSPO expression compared with the CON group (Fig. S4, *Z* > 2.3, *p*_cluster_ < 0.05), while HFA and LFA groups did not differ.

#### Controlling for differences in BMI

An analysis with a subgroup of controls that did not differ from the individuals with ASD in terms of BMI (15 ASD, BMI: 28.9 ± 5.9 (mean ± SD), 13 CON, BMI: 26.2 ± 2.9 (*p* > 0.1, ns)) confirmed our findings (Fig. S5).

### Correlation between [^11^C]PBR28 binding and symptom severity

**Fig. 2:**
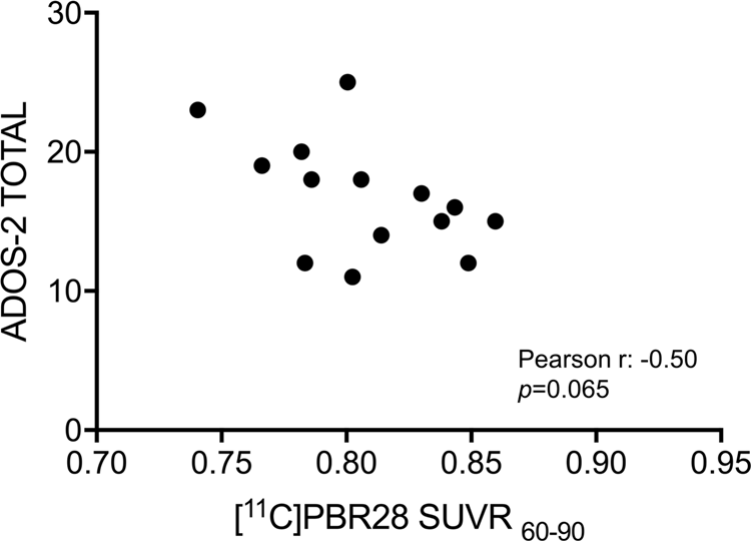
Negative correlation between ADOS-2 total scores and [^11^C]PBR28 SUVR_60–90_ in the region in which the ASD group show a decrease in relative TSPO expression compared with the CON group, reflecting that individuals with more severe autism symptoms tend to show lower relative TSPO expression in those brain regions. ADOS-2 total scores are calculated as the sum of ADOS-2 social affect and ADOS-2 restrictive repetitive behaviors.

A post hoc correlation analysis of ADOS-2 total scores [56], calculated as the sum of the two ADOS-2 domains: ADOS-2_social affect_ and ADOS-2_restricted repetitive behaviors_, and [^11^C]PBR28 SUVR_60–90_ in the brain area that showed a decrease in ASD compared with CON (*p*_FWE_ < 0.05) revealed a trend for individuals with higher autism symptom severity to exhibit lower [^11^C]PBR28 SUVR_60–90_ (Pearson’s *r* = −0.50, *p* = 0.065, Fig. 2) for the 14 participants with ASD with ADOS-2 scores.

### Long-term stability of [^11^C]PBR28 SUVR

**Fig. 3:**
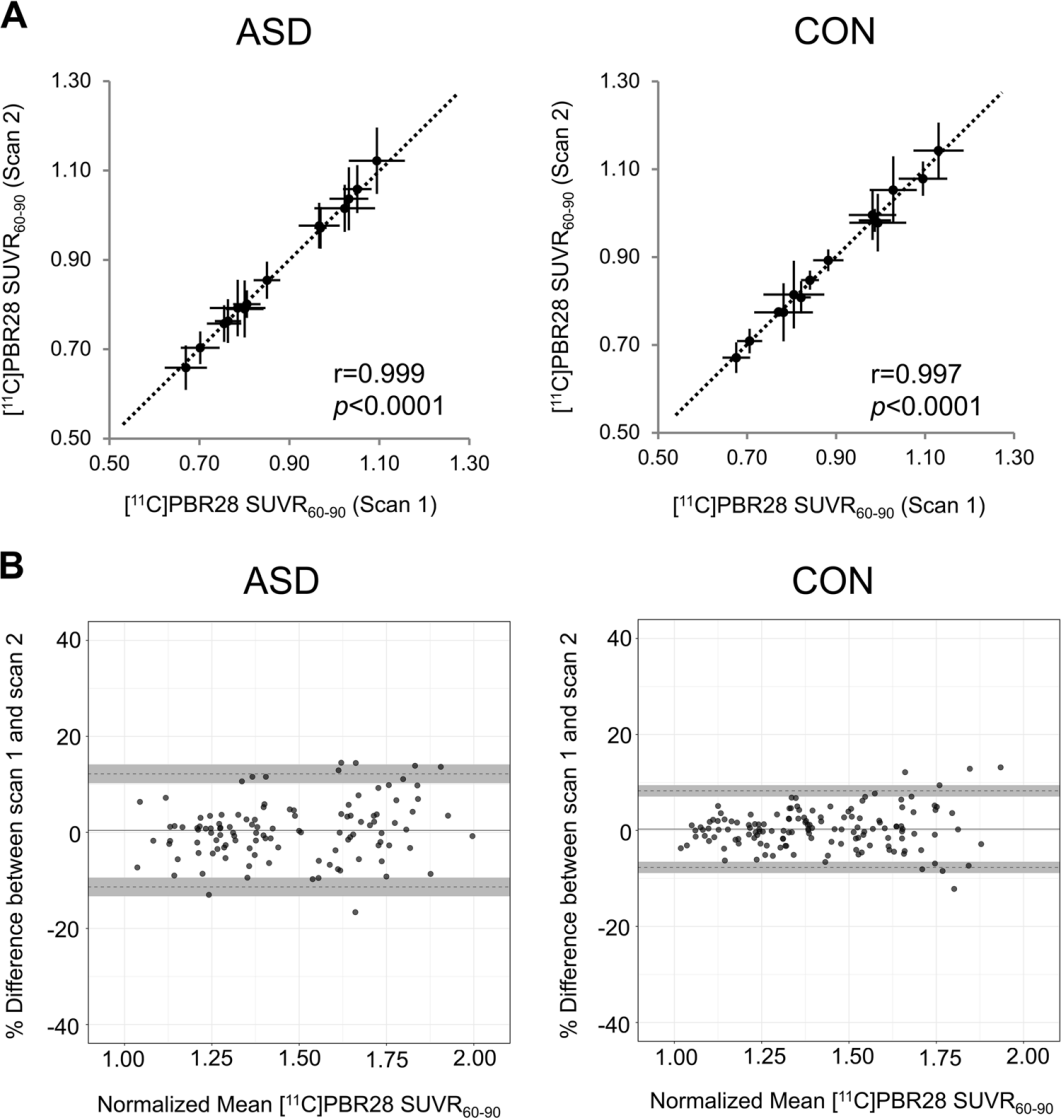
Analysis of long-term [^11^C]PBR28 SUVR stability. **a** Correlation plots for ASD and CON illustrate the stability of [^11^C]PBR28 SUVR_60–90_ over a median rescan interval of 3.6 months. [^11^C]PBR28 SUVR_60–90_ for 14 volumes of interest were assessed for both time points and Pearson’s *r* shows a strong correlation between [^11^C]PBR28 SUVR_60–90_ at those two time points. The dotted line represents the identity line. **b** Bland–Altman plots of interscan variability. To more easily compare ASD and CON, [^11^C]PBR28 SUVR_60–90_ values were normalized to a range of 1–2. The solid line represents the mean difference between scan 1 and scan 2, and points represent each ROI from each subject. The dotted lines are the upper and lower limits and the shaded regions define the 95% CI of the upper and lower limits.

The median rescan interval across both groups was 3.6 months (ASD: median 4.5 (range 3.3–7.9 months), CON: 3.1 (range 2.6–5.2 months)). [^11^C]PBR28 uptake was stable over this time in both ASD and CON. See Table S1 for mean ± SD for ASD and CON groups for the 14 ROIs for both time points as well as percent signal change over time. [^11^C]PBR28 SUVR between the two scans was highly correlated (Pearson’s *r*: 0.999 in ASD, Pearson’s *r*: 0.997 in CON, both *p* < 0.0001, Fig. 3a). For both groups, high reproducibility was found with nearly all regions falling within the CR limits. In the ASD group, mean bias was 0.42 ± 6.01% and 0.28 ± 4.07% in the CON group, Fig. 3b.

## Discussion

Putative roles for TSPO include steroidogenesis, apoptosis, neuroimmune mechanisms/(neuro)inflammation, energy production, mitochondrial dysfunction, and oxidative stress [57]. A change in TSPO expression as assessed by PET in ASD individuals may therefore reflect abnormality in any of those processes. In our study, males with ASD exhibited lower regional TSPO expression compared with age-matched control males, with a trend for higher autism symptom severity to be associated with lower TSPO expression. We further observed the stability of the relative TSPO expression across brain regions over a period of several months in both individuals with ASD and controls, both of which were expected given reports of the stability of longitudinal TSPO expression in healthy controls using SUVR_(cerebellum)_ [58], and the fact that ASD is a chronic condition with a stable disease phenotype in adulthood. Lower TSPO expression was observed in brain regions associated with sociocognitive processes, including the temporal lobe and insula, which have repeatedly shown abnormal activation in ASD individuals in functional MRI (fMRI) studies. The colocalization of altered TSPO expression in areas known to show abnormal activation in fMRI studies in ASD is notable, while the lower TSPO expression in ASD compared with CON is perhaps surprising given autism-related inflammation hypotheses, if TSPO is being interpreted as a proxy for an inflammatory signal. However as previously noted, this interpretation may not be appropriate as the signal may relate to other physiological roles of TSPO [57]. Under physiological conditions, TSPO expression in the healthy brain is low, while under inflammatory conditions (as they occur during injury or neurodegenerative disease) it is widely upregulated in microglia and astrocytes [23, 59] as well as on the endothelial layer of blood vessels [60]. TSPO can also be expressed in some neurons [23]. TSPO’s role in mitochondrial function and how its expression may change in disease is less well understood (reviewed in ref. [61]). Abnormal mitochondrial function could be driving the signal difference observed here between individuals with ASD and controls. We observed decreased [^11^C]PBR28 uptake in the temporal lobe, where mitochondrial dysfunction has previously been observed in individuals with ASD [4, 21, 22]. It is possible that mitochondrial dysfunction or mitophagy (degradation of mitochondria by autophagy) is occurring in brain areas with lower [^11^C]PBR28 uptake in ASD and that this process is more pronounced in more severely affected individuals, as in vivo TSPO levels in the adult tended to correlate with symptom presentation. In rodents, abnormalities in microglia autophagy have been associated with synaptic defects and social deficits [62]. Interestingly, recent PET studies with second-generation TSPO radiotracers have reported a decrease in expression or the lack of a group difference in individuals with schizophrenia compared with controls (refs. [63–67] for volume of distribution (*V*_T_) data). Similar to ASD, TSPO overexpression had originally been predicted in schizophrenia based on preclinical models and increased peripheral cytokines in patients, but was not seen by in vivo PET imaging [63–66]. Notwithstanding the difficulties of modeling autism-implicated mechanisms in animal models, it is interesting to note that in the context of the maternal poly(I:C)-induced immune activation model, a commonly used model in the context of autism and other psychiatric disorders such as schizophrenia, decreases in TSPO expression have been observed [68].

As emphasized by others, it is crucial to consider TSPO expression within the disease-specific context [69]. Given TSPO’s multiple putative physiological functions, it is entirely possible that different mechanisms are at play in different diseases, and the mechanisms behind changes in TSPO expression in the context of ASD (and schizophrenia) are likely different from those underlying TSPO expression changes in a neurodegenerative disease, such as amyotrophic lateral sclerosis (ALS) [33, 34, 40] or Alzheimer’s disease [58], where increases are observed. A challenge inherent to PET imaging of TSPO is that TSPO is expressed in different cell types including microglia, astrocytes, vascular endothelial cells, and neurons. While the increases in TSPO expression are generally easier to interpret and appear to be largely driven by glial cells, decreased TSPO expression as observed here in ASD could reflect changes in both glia, endothelial cells or neurons, and will need to be determined in the disease-specific context.

To the best of our knowledge, no other study has so far investigated TSPO in ASD using a second-generation radiotracer. A previous study with the radiotracer [^11^C]PK1195 reported an increase in TSPO expression in ASD in several brain regions [24], but [^11^C]PK11195 has a much lower ratio of specific to nonspecific binding ratio than [^11^C]PBR28 (~80× in primates) [70]. Furthermore, as pointed out by others, the authors of this study used an unconventional data analysis approach, which relied on using data from the control group to normalize the data for both controls and individuals with ASD [71]. One small post mortem study with eight individuals with ASD, which investigated TSPO gene expression in a limited number of brain regions reported no change in the motor cortex or thalamus and a modest increase in the anterior cingulate cortex in ASD, along with a decrease of numerous other mitochondria-related genes [25]. In addition to looking only at genes and not proteins, the study differed from ours in that it included participants across a wide age range (children and adults) and included both males and females [25]. Post mortem studies that measured microglia numbers or microglial volume in ASD found no difference in the amygdala, but an increase in insula, visual cortex, and dorsolateral prefrontal cortex [72–75]. However, it is important to note that those studies did not investigate TSPO, but other histopathological markers of inflammation (microglial numbers/volume). Furthermore, those studies did not exclude for comorbid epilepsy, which is associated with neuroinflammation and is associated with increased in vivo TSPO expression [30, 76], included a wide age range and were limited to a few ROIs. Those studies are therefore difficult to directly compare with the current work, particularly given that they did not investigate TSPO. As previously mentioned, one clinical trial has investigated the efficacy of a nonsteroidal anti-inflammatory drug (celecoxib) in autism [18]. However, the interpretation of this study is challenging since drug response mechanisms were not decoupled from clinical response. Thus, the outcome cannot determine whether the drug modified the appropriate biological process (measured through a response biomarker) and whether the biological hypothesis was valid. This may be addressed in the future with PET imaging in the context of clinical trials to ensure target engagement. Furthermore, the treatment trial was conducted in children with autistic disorder, whereas our imaging study was conducted in adults with ASD. It may be that there are differences in the status of disease mechanism at different points across development in ASD.

The TSPO decreases we observed were not due to atrophy in males with ASD compared with controls, as volumes of affected brain areas were not abnormal in ASD, and the effect remained present after PVC. Our study does, however, have some potential limitations. Given the ethical considerations when enrolling low-functioning participants who require surrogate consent, this study was conducted without collecting any arterial line blood data and by using SUVR_60–90_ as the primary outcome measure. We have previously shown that with [^11^C]PBR28 whole-brain normalizations, we were able to detect significant increases in TSPO activation in chronic pain [35, 77], primary lateral sclerosis [38], and ALS [33, 40], with strong regional overlap between [^11^C]PBR28 SUVR_60–90_ and *V*_T_ and/or *V*_T_ ratio (DVR) [32, 77]. Future work using arterial sampling will focus on determining a suitable pseudo-reference region in ASD, an approach that has been successfully applied and validated for [^11^C]PBR28 for other disease populations [32, 78]. While there is a need to study individuals across the autism spectrum [28], their inclusion comes with the additional challenge of comorbid intellectual disability, potentially complicating the interpretation of results. Future studies could include control groups that are IQ-matched, which, however, comes with its own limitations as it would require including a control group with intellectual disabilities. Also, although participants were not included if they were on benzodiazepines, anti-inflammatory, or immune-modulatory drugs, participants with ASD were not drug free, as many were prescribed antidepressants, anxiolytics, atypical antipsychotics, or stimulants as part of their clinical care, which could potentially affect [^11^C]PBR28 uptake [79, 80]. Studies of much larger sample size are required to address the question of effects of specific medication on [^11^C]PBR28 uptake in ASD and other diseases. In order to study TSPO expression in a more homogenous group and given known sex differences in glial biology and the higher prevalence of ASD in males, this work focused on TSPO expression in males. Ongoing work in our group is assessing TSPO in females with ASD in order to investigate whether there are any sex differences. This may be particularly interesting given that immune cells mediate brain masculinization early in life [81], which may confer sexually dimorphic vulnerability in a neurodevelopmental disorder such as ASD.

In summary, abnormal TSPO expression was observed in adult males with ASD, and the pathophysiological meaning behind the TSPO decrease still remains to be determined and cannot yet be attributed to a given mechanism or cell type. Reverse translation in the disease-specific context will be required to determine what changes in TSPO expression reflect. Studies conducted in other disease contexts have had success combining in vivo TSPO PET imaging and post mortem histological characterization [82]. Studies with a focus on post mortem tissue analysis will need to be conducted, and will benefit from the information obtained through in vivo TSPO imaging in terms of selecting brain regions to investigate. Given the observed decreases in TSPO expression, histological investigation of mitochondrial function-related processes should be emphasized.

## Supplementary information

Figure S1

Figure S2

Figure S3

Figure S4

Figure S5

Table S1

Legends for SI Figures and SI Table

## Data Availability

Custom codes for MR–PET data processing are available from the corresponding authors NRZ and JMH upon reasonable request.
