# Supplementary figures and images for: [11C]PBR28 MR–PET imaging reveals lower regional brain expression of translocator protein (TSPO) in young adult males with autism spectrum disorder
Source: Mol Psychiatry. 2020 Feb 19;26(5):1659–69. doi: 10.1038/s41380-020-0682-z (PMC8159742; doi:10.1038/s41380-020-0682-z)

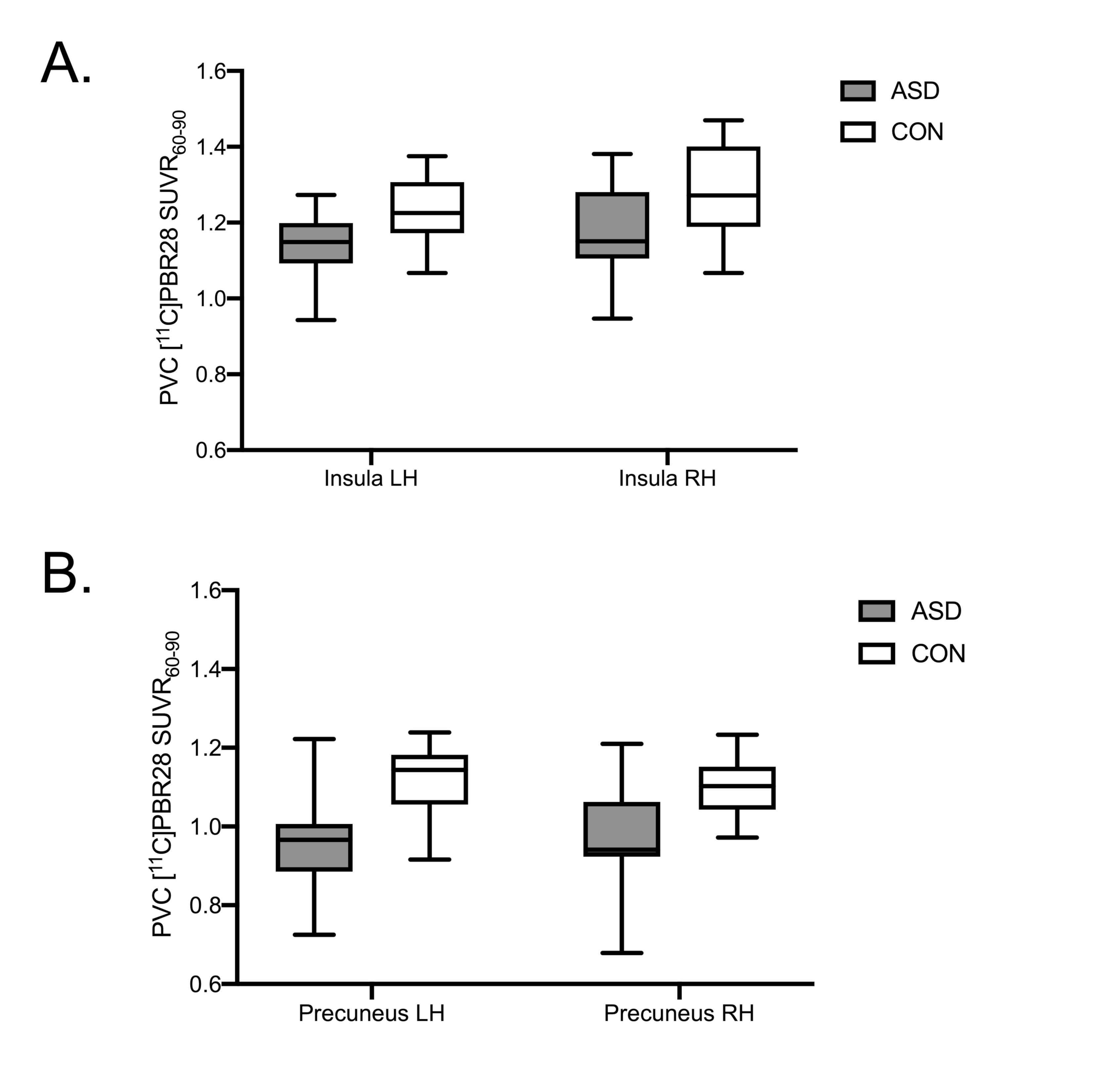

Supplement: Supplementary file 2 — Figure S2 [file 41380_2020_682_MOESM2_ESM.tif]

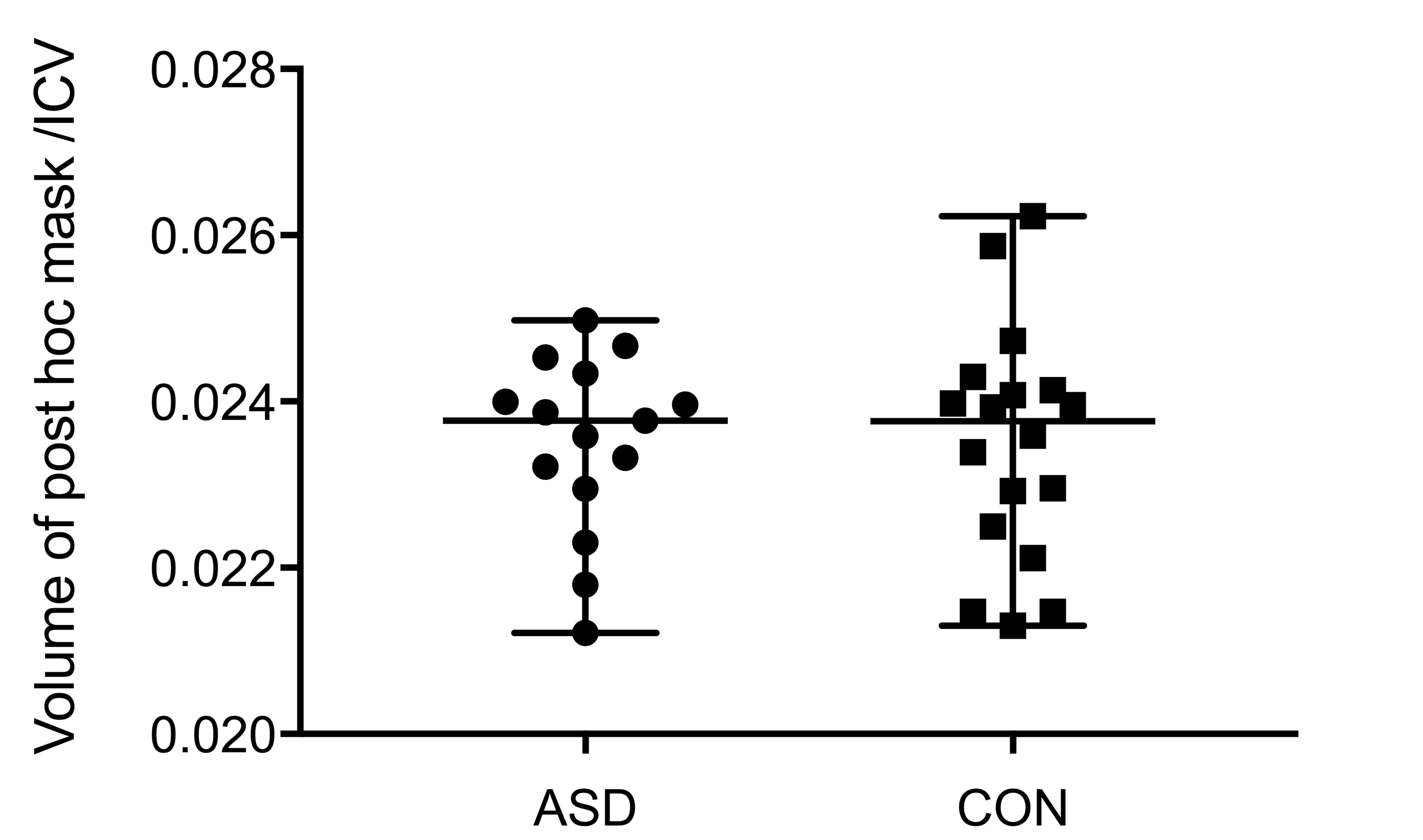

Supplement: Supplementary file 3 — Figure S3 [file 41380_2020_682_MOESM3_ESM.tif]
