# Supplementary material for: [11C]PBR28 MR–PET imaging reveals lower regional brain expression of translocator protein (TSPO) in young adult males with autism spectrum disorder
Source: Mol Psychiatry. 2020 Feb 19;26(5):1659–69. doi: 10.1038/s41380-020-0682-z (PMC8159742; doi:10.1038/s41380-020-0682-z)
Supplement: Supplementary file 6 — Table S1 [file 41380_2020_682_MOESM6_ESM.docx]

SI Table 1

| **ROIS** | **FRO** | **PAR** | **TEMP** | **OCC** | **INS** | **CING** | **CAU** | **PUT** | **PAL** | **THAL** | **HP_pHP** | **AMY** | **CB** | **WM** |
| --- | --- | --- | --- | --- | --- | --- | --- | --- | --- | --- | --- | --- | --- | --- |
| **ASD, scan 1**  **Mean ± SD** | 0.7±0.04 | 0.67±0.05 | 0.75±0.04 | 0.80±0.04 | 0.85±0.03 | 0.81±0.03 | 0.76±0.03 | 0.97±0.01 | 1.05±0.03 | 1.09± 0.06 | 0.97±0.05 | 1.03±0.04 | 1.02± 0.07 | 0.78±0.06 |
| **ASD, scan 2**  **Mean ± SD** | 0.7±0.04 | 0.66±0.05 | 0.76±0.04 | 0.79±0.06 | 0.85±0.04 | 0.80±0.03 | 0.76±0.05 | 0.97±0.05 | 1.06±0.03 | 1.12± 0.07 | 0.98±0.05 | 1.04±0.07 | 1.02±0.05 | 0.79±0.06 |
| **Percent signal difference**  **Mean ± SD** | 0.36± 3.77 | -1.57±4.66 | 0.35±1.39 | -1.38±3.16 | 0.55±3.89 | -0.44±4.77 | -0.02±5.63 | 0.25±3.83 | 0.72±4.40 | 2.52±3.44 | 1.20±5.40 | 0.43±4.91 | -0.51±5.66 | 1.00±3.66 |
| **CON, scan 1 Mean ± SD** | 0.71±0.03 | 0.68±0.03 | 0.77±0.01 | 0.82±0.02 | 0.88±0.03 | 0.84±0.02 | 0.78±0.07 | 0.99±0.04 | 1.1±0.05 | 1.13±0.06 | 0.98±0.05 | 1.03±0.05 | 0.99±0.06 | 0.8±0.07 |
| **CON, scan 2 Mean ± SD** | 0.71±0.03 | 0.67±0.04 | 0.78±0.01 | 0.81±0.04 | 0.89±0.02 | 0.85±0.02 | 0.77±0.07 | 0.98±0.03 | 1.08±0.04 | 1.14±0.06 | 1.00±0.06 | 1.05±0.08 | 0.98±0.07 | 0.81±0.08 |
| **Percent signal difference**  **Mean ± SD** | 0.52±2.16 | -0.64±2.52 | 0.60±1.14 | -1.61±2.25 | 1.24±1.99 | 0.79±2.78 | -0.93±2.77 | -0.31±2.07 | -1.41±2.95 | 1.16±3.95 | 1.47±2.39 | 5.65±3.09 | -1.53±2.33 | 1.16±2.36 |

Percent signal change between the two time points was calculated across subjects and then averaged. Abbreviations: Frontal (FRO), parietal (PAR), temporal (TEMP), occipital (OCC), insula (INS), cingulate (CING), caudate (CAU), putamen (PUT), pallidum (PAL), thalamus (THAL), hippocampus/parahippocampal gyrus (HP_PHP), amygdala (AMY), cerebellum (CB), white matter (WM).
