# Supplementary material for: [11C]PBR28 MR–PET imaging reveals lower regional brain expression of translocator protein (TSPO) in young adult males with autism spectrum disorder
Source: Mol Psychiatry. 2020 Feb 19;26(5):1659–69. doi: 10.1038/s41380-020-0682-z (PMC8159742; doi:10.1038/s41380-020-0682-z)
Supplement: Supplementary file 7 — Legends for SI Figures and SI Table [file 41380_2020_682_MOESM7_ESM.docx]

**Legends for SI Figures and SI Table**

**Figure S1:** Statistical difference in [^11^C]PBR28 SUVR_60-90_  between ASD (N=8) vs. controls (N=10) who underwent a second scan. Individuals with ASD showed decreased [^11^C]PBR28 SUVR_60-90_ in the left supramarginal gyrus, the left lateral occipital cortex and bilateral precuenus/posterior cingulate cortex. No region showed an increase in [^11^C]PBR28 SUVR_60-90_  in  ASD compared to CON.

**Figure S2:** [^11^C]PBR28 SUVR_60-90_ with partial volume correction (PVC) in the insula of the left hemisphere (LH) and right hemisphere (RH) (Figure S1A) and the left and right precuneus (Figure S1B) in subject space in individuals with ASD and CON. A 2x2 ANOVA shows a significant group effect for the insula, (F(1,62)=14.09, *p<*0.0005) and for the precuneus (F(1,62)=29.88, *p<*0.0001), with ASD showing decreased PVC [^11^C]PBR28 SUVR_60-90_ compared to CON. Data are shown as median and range.

**Figure S3:** The volume of the region showing lower [^11^C]PBR28 SUVR_60-90_ in ASD compared to controls in the voxelwise analysis (post hoc mask) normalized by the estimated total intracranial volume (ICV), is not significantly different between individuals with ASD and CON (Mann-Whitney, *p*=0.87, *ns*). Data are shown as median and range.

**Figure S4:** Statistical difference in [^11^C]PBR28 SUVR_60-90_  between low-functioning ASD (LFA) (N=6) vs. controls (N=18) (top panel), and between high-functioning ASD (HFA) (N=9) vs. controls (N=18) (bottom panel). No region showed an increase in [^11^C]PBR28 SUVR _60-90_  in  LFA or HFA compared to CON. A voxelwise analysis comparing LFA and HFA showed that they were not significantly different from each other.

**Figure S5:** Statistical difference between [^11^C]PBR28 SUVR_60-90_  in ASD (N=15) compared to a subset of healthy controls (N=13), which do not show a difference in BMI compared to the ASD group (*p*>0.1, *ns*). No region showed an increase in [^11^C]PBR28 SUVR_60-90_  in  ASD compared to CON.

**Table S1:** Percent signal change between the two time points was calculated across subjects and then averaged. Abbreviations: Frontal (FRO), parietal (PAR), temporal (TEMP), occipital (OCC), insula (INS), cingulate (CING), caudate (CAU), putamen (PUT), pallidum (PAL), thalamus (THAL), hippocampus/parahippocampal gyrus (HP_PHP), amygdala (AMY), cerebellum (CB), white matter (WM).
